# Mesenchymal Stromal Cells Support the Viability and Differentiation of Follicular Lymphoma-Infiltrating Follicular Helper T-Cells

**DOI:** 10.1371/journal.pone.0097597

**Published:** 2014-05-16

**Authors:** Michael T. Brady, Shannon P. Hilchey, Ollivier Hyrien, Stephen A. Spence, Steven H. Bernstein

**Affiliations:** 1 James P. Wilmot Cancer Center, University of Rochester Medical Center, Rochester, New York, United States of America; 2 Department of Biostatistics and Computational Biology, University of Rochester Medical Center, Rochester, New York, United States of America; University of Texas, MD Anderson Cancer Center, United States of America

## Abstract

The biology of follicular lymphoma (FL) is largely dictated by the immune-effector and stromal cells that comprise its tumor microenvironment. FL-infiltrating T-cell populations that are thought to be fundamental to FL biology are follicular helper T-cells (TFH), follicular regulatory T-cells (TFR), a recently described population that regulates TFH activity, and regulatory T-cells (Treg). These T-cell populations have dynamic interactions with mesenchymal stromal cells (MSCs) in the tumor microenvironment. Whereas MSCs have been shown to support FL B-cell and Treg viability, their effects on FL-infiltrating TFH and TFR cells have not been described. Herein we show that MSCs support the viability of FL-infiltrating TFH and TFR, as well as Tregs, in part through an IL-6-dependent mechanism. We further demonstrate that MSCs mediate TFH to TFR conversion by inducing the expression of FoxP3 in TFH cells, demonstrating for the first time that human TFR can be derived from TFH cells. Given that the balance of TFH and TFR populations likely dictate, in part, the biology of this disease, our data support the potential for targeting MSCs as a therapeutic strategy.

## Introduction

Follicular lymphoma (FL) is an indolent lymphoma having a natural history, which is dictated, in part, by the interactions between the malignant B-cells and the non-malignant cells comprising its microenvironment. One component of this microenvironment, which has been shown to support the viability and induce the chemotherapeutic resistance of FL B-cells, are Mesenchymal Stromal Cells (MSCs) [1], [2]. MSCs support FL B-cell viability through their expression of adhesion molecules and integrins, which provide survival signals to the FL B-cells upon binding to their cognate receptors, as well as by their elaboration of pro-survival cytokines such as IL-6 and BAFF [1], [3], [4]. Further, MSCs may contribute directly to lymphomagenesis, through their production of vascular endothelial growth factor, for example, or indirectly through their effects on the viability and differentiation of the FL-infiltrating CD4^+^ helper T-cells (Th) [5], [6].

Gene expression and immunohistochemistry studies demonstrate that FL-infiltrating Th cells impact FL biology and show a correlation between the number and anatomical location of distinct Th cell populations with patient survival [7]. One such Th cell population is regulatory T-cells (Treg), a T-cell subset which suppresses both effector T-cell priming and cytotoxicity and whose differentiation is controlled by the FoxP3 transcription factor. We have previously shown that FL-infiltrating Tregs potently inhibit the proliferation and cytokine production of FL-infiltrating T-cells [8]. MSCs have been shown to induce the differentiation of naïve T-cells to Tregs, and as such MSCs may modulate FL biology, in part, through their support of Treg differentiation [9].

Follicular helper T-cells (TFH) are another Th population that we, and others, have shown to be present in FL involved lymph nodes [10]–[12]. TFH cells comprise a greater proportion of CD3^+^ T-cells in FL nodes compared to that seen in normal lymph nodes [10]. TFH cells express the Bcl-6 transcription factor and support the survival and differentiation of normal germinal center (GC) B-cells [13]. While less is known about their effects on FL B-cells, recent studies suggest that TFH cells support FL B-cell viability through their generation of IL-4 and their expression of CD40 ligand [10]. TFH support of normal GC B-cell viability is inhibited by the recently characterized T-follicular regulatory cells (TFR), a T-cell population characterized by their dual expression of FoxP3 and Bcl-6 and one which we and others have shown to be present in the FL microenvironment [10], [11], [14]–[16].

It is likely the balance between the TFH and TFR cells, which regulates FL B-cell viability. Therefore it was of great interest to determine whether MSCs in the FL microenvironment regulate the survival and differentiation of TFH and TFR cells, as they do Tregs, and to gain further insight into how MSCs modulate FL biology. We demonstrate that: a) MSCs support the viability of FL-infiltrating TFH and TFR in part, through an IL-6-dependent mechanism and b) MSCs promote the differentiation of TFH to TFR by inducing their expression of FoxP3.

## Materials and Methods

### Patient Samples

Follicular lymphoma lymph nodes (LN), bone marrow (BM) aspirates and tonsils (TN) were obtained from patients undergoing routine biopsy or tonsillectomy. Biopsy tissues were obtained from the University of Rochester/Arizona Cancer Center SPORE Tissue Resource Core and tonsil tissue was obtained from the Strong Memorial Hospital Surgical Pathology Laboratory. All specimens were acquired under a University of Rochester Institutional Review Board approved protocol. Informed written consent was obtained in accordance with the Declaration of Helsinki.

### Mesenchymal Stromal Cells

MSCs were isolated from single cell suspensions (SCS) of TN and FL LN and BM tissues. One million cells/cm^2^/mL were plated in RPMI 1640 with 10% FBS (Life Technologies), penicillin/streptomycin (Cellgro), (R10) supplemented with 12.5 ng/mL basic fibroblast growth factor (R&D Systems). Non-adherent cells were discarded after 2 days of culture. Adherent cells were expanded until confluent, replacing media every 3 days. MSCs were used from passages 2–8.

Adipogenic induction was achieved by culturing confluent MSCs in adipogenic induction/maintenance media according to the manufacturer’s protocol (Lonza). Cells were fixed in 10% formalin and stained with an Oil Red O solution (Sigma) to visualize lipid vacuoles. Osteoblast differentiation was induced by culture for 3 weeks in R10, supplemented with 10 µM dexamethasone, 0.1 mM ascorbic acid and 10 mM β-glycerophosphate (Sigma). Early osteoblast differentiation was assessed by alkaline phosphatase staining after fixation and incubation with a solution containing Naphthol AS-MX-phosphate and Fast Red LB salts (Sigma).

### Cell Co-cultures

FL-SCS or FL T-cell subsets (1.5e^6^ cells/mL) were cultured with media alone or with confluent MSCs in R10 with or without Transwell plates. After 48 hrs, supernatants and cells were collected for analysis. In some experiments B-cells were depleted from FL-SCS with CD19 Dynabeads (Invitrogen) and depletion was confirmed by flow cytometry. In some experiments CD3^+^CD4^+^ T-cells were sorted from FL-SCS samples using a FACSAria cytometer (BD Biosciences).

Supernatants from 48 hr co-cultures were analyzed for IL-6 and IL-21 proteins using Ready-SET-Go Human ELISA kits (eBioscience). In some experiments neutralizing antibodies to IL-6 and/or IL-21 (eBioscience) were added to a final concentration of 5µg/mL at the start of culture.

TFH were sorted from FL-SCS using a FACSAria and subsequently analyzed by flow cytometry for intracellular proteins or placed in culture with or without MSCs as described below. After 48 hrs the TFH cells were analyzed by flow cytometry.

### Flow Cytometry

MSCs were analyzed using the following antibodies: CD73-PE, CD105-PE, CD14-PE, CD45-FITC, CD90-APC (BD Biosciences), CD106-Pe-Cy5, CD34-Pe-Cy5 (BD Biosciences), HLA-DR-Qdot-605 (Invitrogen), mouse mAb specific to FAP (eBioscience) and PE-conjugated anti-mouse (BD Biosciences). Isotype-matched, fluorochrome-conjugated, mAb were used as negative controls.

Cultured SCS were stained, data acquired and analyzed as previously described using the following antibodies [11]; Surface: CD3-Qdot-605, CD4-Qdot-655 (Invitrogen), CXCR5-Alexa-488, CD25-APC-Cy7, PD-1-Brilliant Violet-421; Intracellular: Bcl-6-PE-CF594, active caspase-3-PE (BD Biosciences), and FoxP3-PE-Cy7 (eBioscience).

FL T-cell subsets were flow cytometrically sorted using the following markers; T-cell: DAPI^−^CD3^+^CD4^+^CD19^−^; TFH: Propidium iodide^−^CD3^+^CD4^+^CXCR5^+^PD-1^+^CD25^−^. Following the sort, an aliquot of TFH were permeabilized and stained with Bcl-6 and FoxP3 to confirm the TFH phenotype. TFH cells were defined as CD3^+^CD4^+^CXCR5^+^PD-1^+^CD25^−^Bcl-6^+^; TFR were defined as CD3^+^CD4^+^CXCR5^+^PD-1^+^CD25^+^Bcl-6^+^FoxP3^+^ and Tregs were defined as CD3^+^CD4^+^CD25^+^FoxP3^+^.

### Quantitative RT-PCR

Total RNA was extracted and amplified from isolated MSCs and used to quantify the expression of *GAPDH*, *IL-6* and *IL-21* as we have previously published [11].

### Statistical Analysis

The goal of the analyses was to compare the proportion of Th subsets. These variables (subsets) were analyzed using one-way mixed ANOVA models with “culture condition” treated as a factor. Models included two crossed random intercepts to describe between-day and between-patient variability and assumed the error terms and random intercepts were normally distributed. Assumptions were assessed using normal probability plots and histograms of conditional and unconditional residuals. In the event normality assumptions did not appear to be met, our analyses were performed after applying a suitable transformation (logarithmic) to the data such that the normality assumptions would appear satisfied. Point estimates of the difference in population size across culture conditions are reported without transformation. Comparison of the sizes of cell subsets were performed using Wald tests. All tests were two-sided and conducted at the 5% significance level (probability of type-1 error). Analyses were performed using the SAS statistical package, version 9.13 (SAS Institute).

Data on the TFH, Treg and TFR populations from the depleted B-cell experiments were analyzed using two-way mixed ANOVA. The models included the presence of MSC (yes vs. no), B-cells (depleted vs. non-depleted) and their interaction as factors, as well as random intercepts to describe inter-patient variation. The data were preliminary log-transformed to correct for skewness. Absence of interactions in the models would mean that the effect of MSC on cell frequency is identical in B-cell depleted and in B-cell non-depleted conditions. It would also mean that the effect of B-cells on cell frequency is consistent whether MSC were present or absent from the culture. The main effect for MSC describes the change in mean cell frequency when MSC are added in the culture, and similarly for the main effect of B-cells. If the interaction is absent, the effect of the MSC factor is the same regardless of the levels of the B-cell factor, and vice versa, as described by their main effects. We assessed the significance of the main effect of each factor (MSC and B-cell depletion) and of their interaction using F-tests, and report associated p-values. The data collected for the isolated T-cell experiments were analyzed using the same approach.

## Results

### Characterization of Lymphoid-derived Stromal Cells

MSCs isolated from healthy donor tonsils (TN) and FL-involved tissues displayed typical fibroblast morphology and expressed surface proteins characteristic of MSCs (CD73, CD90, CD105, CD106 and fibroblast activation protein (FAP)), while lacking expression of the lineage specific markers CD14, CD34, CD45 and HLA-DR (Fig. S1A). TN and FL-derived stromal cells were able to differentiate into adipocytes and osteoblasts under the appropriate conditions (Fig. S1B). Taken together these properties fulfill the criteria used for characterizing such cells as MSCs [17]. TN and FL-derived MSCs both met the criteria for characterization as MSCs and both had similar effects on the viability and differentiation of Th cell populations. It was technically difficult however to propagate MSC from FL lymph nodes or bone marrow and could only be accomplished in a limited number of samples. Taken together, in order to ensure consistency throughout the studies and to avoid confounding factors associated with patient-patient biological variability, the majority of the studies were conducted with TN-MSCs expanded from a single donor. Selected experiments were repeated with FL-MSCs however when such cells could be generated.

### MSCs Support TFH, Treg and TFR Populations

**Figure 1:**
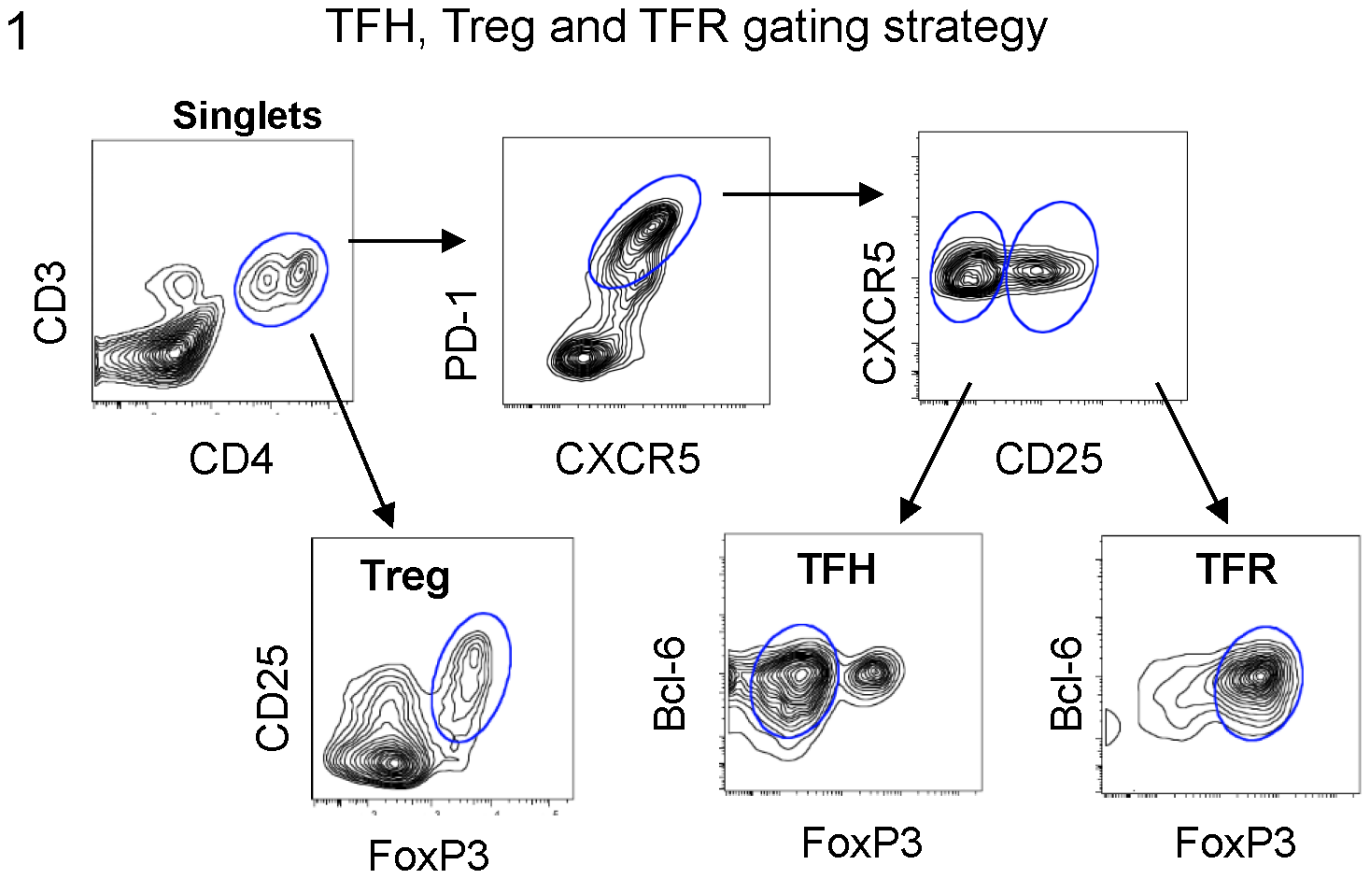
Characterization of FL-infiltrating T-cells by flow cytometry. Shown is an example of the gating strategy used to define TFH, Treg and TFR subsets. Viable lymphocytes were gated according to size, granularity and expression of CD3 and CD4. T-cell subsets were classified as: TFH (CXCR5^+^PD-1^+^CD25^−^Bcl-6^+^FoxP3^−^), Treg (FoxP3^+^CD25^+^) and TFR (CXCR5^+^PD-1^+^CD25^+^Bcl-6^+^FoxP3^+^).

To determine the effect that MSCs have on the viability of FL-infiltrating TFH, Tregs and TFR cells, SCS from FL nodal biopsies were cultured with or without TN-MSCs for 48 hrs. As shown in Fig. 1; TFH cells were defined as CD3^+^CD4^+^CXCR5^+^PD-1^+^CD25^−^Bcl-6^+^; TFR were defined as CD3^+^CD4^+^CXCR5^+^PD-1^+^CD25^+^Bcl-6^+^FoxP3^+^; Tregs were defined as CD3^+^CD4^+^CD25^+^FoxP3^+^.

**Figure 2:**
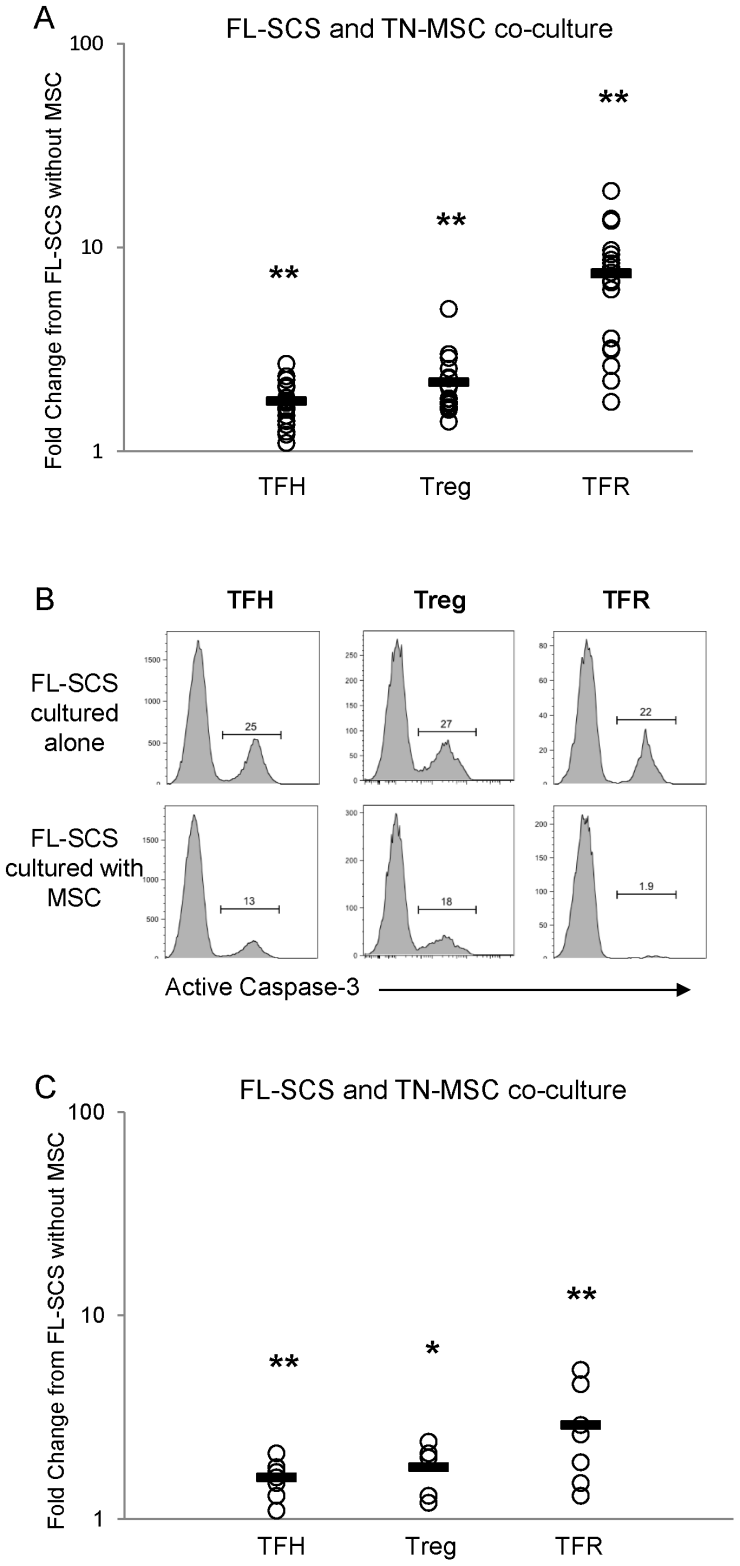
MSCs support FL-infiltrating TFH, Treg and TFR subsets. The proportion of T-cells with phenotypes indicating TFH, Treg and TFR were analyzed by intracellular flow cytometry after culture for 48 hrs in media alone or with TN-MSCs (**A**). Flow cytometry histograms showing reduced active caspase-3 in TFH, Treg and TFR cells after culture with TN-MSCs (**B**). The proportion of T-cells with phenotypes indicating TFH, Treg and TFR were analyzed after 48 hrs of culture with FL BM-derived MSCs n = 2 (**C**). Average fold change from T-cells cultured without MSCs is indicated by solid line, *p<0.05, **p<0.01.

Fig. 2A shows the fold change in the proportion of CD3^+^CD4^+^ T-cells that are a given Th phenotype when FL-SCS were co-cultured with TN-MSCs compared to being cultured without TN-MSCs. When cultured with MSCs there was a significant increase in the proportion of TFH, Tregs and TFR, having a 1.76, 2.19 and 7.47 fold increase, respectively, over that seen in culture without MSCs (p<0.0001) (the actual values of these populations is shown in Table S1). Flow cytometric analysis using quantitative beads revealed that the absolute number of T-cells was reduced across all of these Th subsets after 48 hrs in co-culture with and without MSCs. In addition, when FL-SCS were cultured with MSCs, TFH, Tregs and TFR had reduced levels of active caspase-3 when compared to the same populations cultured without MSCs (Figure 2B). Together, these results suggest that MSCs support TFH, Tregs and TFR cells by inhibiting their apoptosis and supporting their viability rather than by inducing their proliferation or differentiation.

To determine whether MSCs derived from FL BM provided the same support to FL-infiltrating Th populations as did TN-MSCs, FL-SCS were cultured with or without FL BM-derived MSCs for 48 hrs (Fig. 2C). Similar to what was seen with TN-MSC, there was a significant increase in the proportion of T-cells that had a phenotype consistent with TFH, Tregs and TFR when cultured with FL-MSCs, having a 1.6, 1.8 and 2.9 fold increase, respectively, over that seen in culture without MSCs (p = 0.0008, p = 0.0114, p = 0.0034, respectively; Fig. 2C).

Given that these experiments were performed using MSCs derived from a single allogeneic FL BM sample, we next cultured FL-SCS with MSCs derived from autologous FL-involved LN and FL-involved BM tissue to exclude the possibility that the MSC support of the Th subsets is due to an allogeneic specific response. Compared to FL-SCS cultured alone, FL-derived autologous MSCs supported the viability of autologous TFH, Treg and TFR subsets supporting the notion that MSCs derived from either TN, allogeneic or autologous FL nodes or BM similarly supported the viability of TFH, Tregs and TFR cells.

### MSCs Support TFH, Treg and TFR Subsets through Soluble Factors

**Figure 3:**
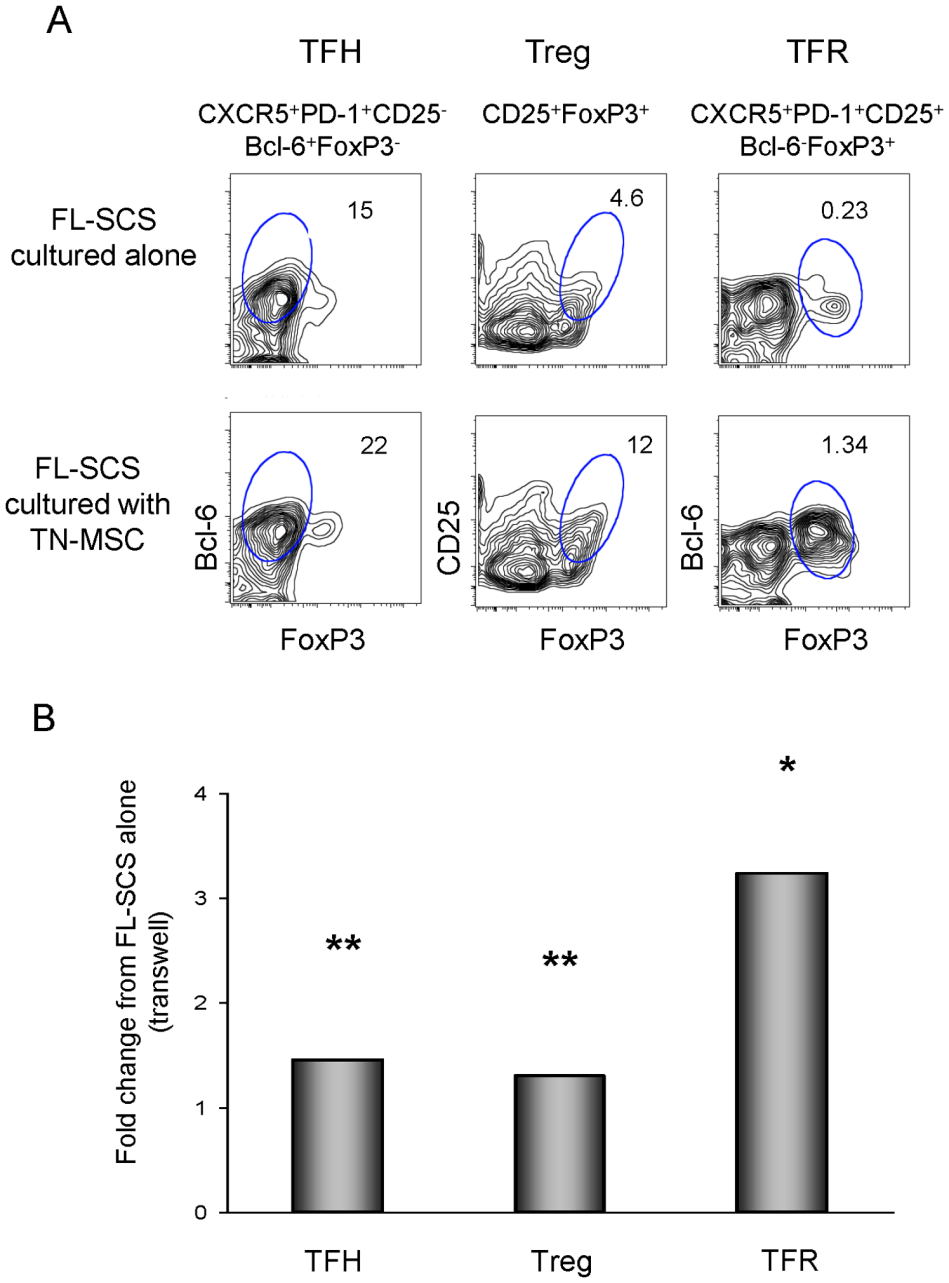
Cell contact is not required for MSC support of T-cell populations. (**A**) Flow cytometry plot depicting increases in the proportion of FL-infiltrating T-cells with phenotypes consistent with TFH, Treg and TFR populations in transwell cultures of FL-SCS and FL-SCS/TN-MSC. (**B**) Bar graph representing average fold change of FL-infiltrating T-cell populations cultured in transwell plates with TN-MSCs compared to T-cell populations cultured in transwell plates without TN-MSCs after 48 hrs (n = 7, *p<0.05, **p<0.01).

We next sought to determine whether the MSC support of TFH, Treg and TFR was contact dependent. FL-SCS were cultured for 48 hrs with or without MSCs separated by a cytokine-permeable transwell membrane. In the example shown in Fig. 3A, when FL-SCS were cultured in transwell plates without MSCs the proportion of CD3^+^CD4^+^ cells that were TFH, Tregs and TFR was 15%, 4.6% and 0.23%, respectively. In contrast, when FL-SCS were cultured with TN-MSCs separated by a transwell membrane the proportion of CD3^+^CD4^+^ cells that were TFH, Tregs and TFR increased to 22%, 12% and 1.34%, respectively. The composite of 7 independent experiments with different FL samples is shown in Fig. 3B. Similar to what was shown in the co-culture experiments without the use of a transwell plate (Fig. 2A), there was a significant increase in the proportion of TFH, Tregs and TFR after co-culture with TN-MSCs in a transwell plate, having a 1.5, 1.3 and 3.2 average fold increase, respectively, over that seen in culture without MSCs (p = 0.0047, p = 0.0014, p = 0.0319). Taken together this data shows that the increased viability of these T-cell subsets upon co-culture with MSCs is due, in part, to soluble factors.

### MSC Support of TFH, Treg and TFR is Independent of B-cells

**Figure 4:**
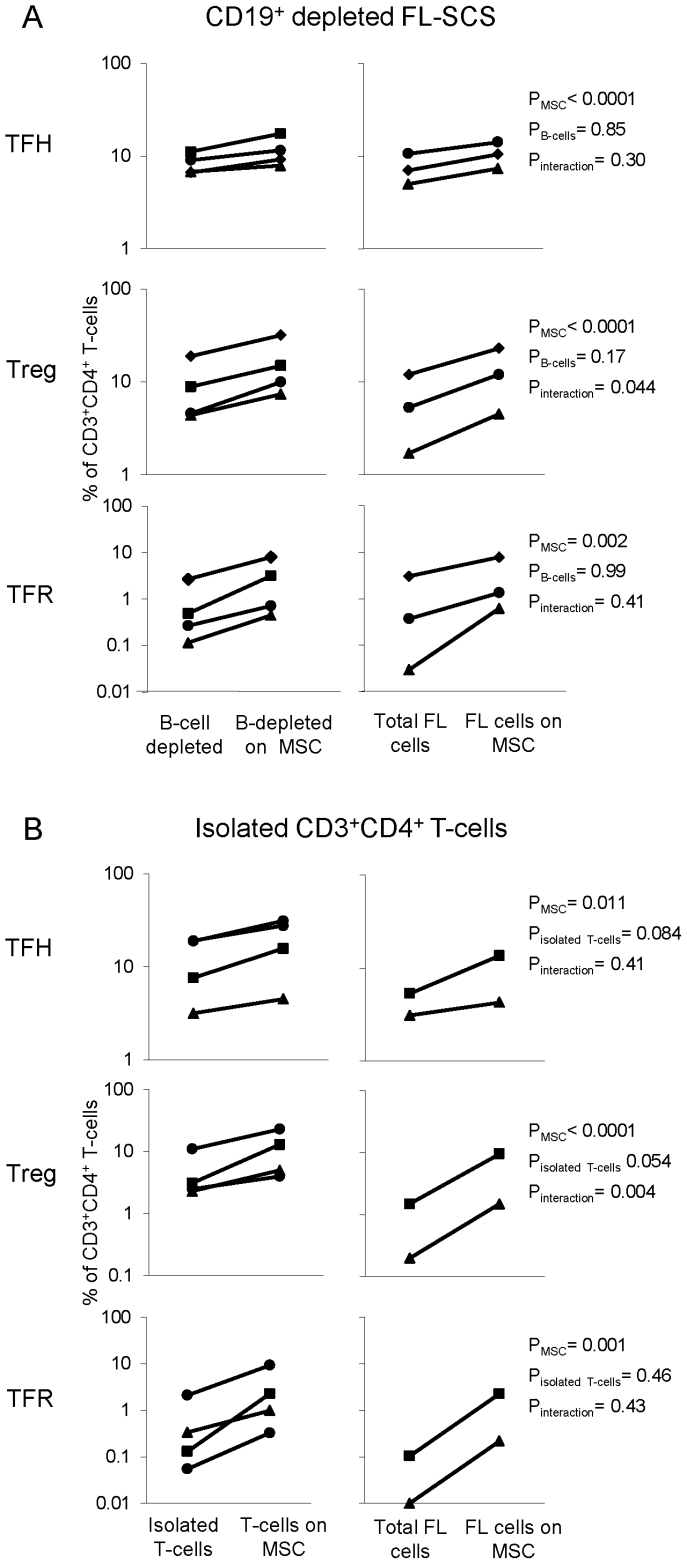
MSCs support FL-infiltrating T-cell subsets in the absence of B-cells. (**A**) CD19^+^ pan B-cell Dynabeads® were used to deplete FL-SCS of B-cells, the remaining cells were cultured alone or with TN-MSCs. Non-depleted FL-SCS cultured in parallel with B-cell depleted experiments served as controls. (**B**) CD3^+^CD4^+^ FACS isolated FL T-cells were cultured alone or with TN-MSCs, Non-depleted FL-SCS cultured with or without TN-MSC served as controls TN-MSCs supported TFH, Treg and TFR populations in purified T-cell cultures.

MSCs have been shown to support normal and malignant B-cell viability and B-cells have been shown to provide survival signals to T-cells. Therefore, one mechanism by which MSCs may support TFH and TFR cell viability is indirectly through their support of B-cells. To determine whether the MSC support of TFH, Tregs and TFR is mediated through its support of FL B-cells, we first measured the effect of TN-MSCs on FL-SCS depleted of CD19^+^ B-cells and compared this to the effects of MSCs on un-depleted FL-SCS controls. This data was then analyzed using a two-way mixed ANOVA including the presence of MSC (yes vs. no), B-cells (depleted vs. non-depleted) and their interaction as factors. As shown in Figure 4A, the presence of MSC had a significant effect on TFH, Treg and TFR frequencies (p =  <0.0001, <0.0001 and 0.002, respectively), whereas the presence or absence of B-cells had no significant effect on TFH, Treg, and TFR frequencies (p = 0.85, 0.17, 0.99, respectively). A borderline interaction between MSC and the presence or absence of B-cells was seen for Tregs (p = 0.044).

These experiments did not, however, exclude the possibility that MSCs were able to support these Th subsets indirectly through their support of another population(s) of cells (other than B-cells) in the highly heterogeneous FL-SCS. To rule out this possibility, we isolated CD3^+^CD4^+^ T-cells from FL-SCS by flow cytometric sorting and cultured these purified T-cells alone or with MSCs for 48 hrs. As shown in Figure 4B, the presence of MSC had a significant effect on TFH, Treg and TFR frequencies (p = 0.011, <0.001 and 0.001, respectively). The presence or absence of other cell populations (ie. non manipulated FL-SCS vs. T-cell isolated FL-SCS) was not significant for TFH, Treg and TFR cells (p = 0.084, 0.054 and 0.46, respectively). An interaction between MSC and the presence or absence of other non-T-cell populations was seen for Tregs (p = 0.0043).

### MSC Secreted Interleukin-6, in Part, Supports T-cell Subsets

**Figure 5:**
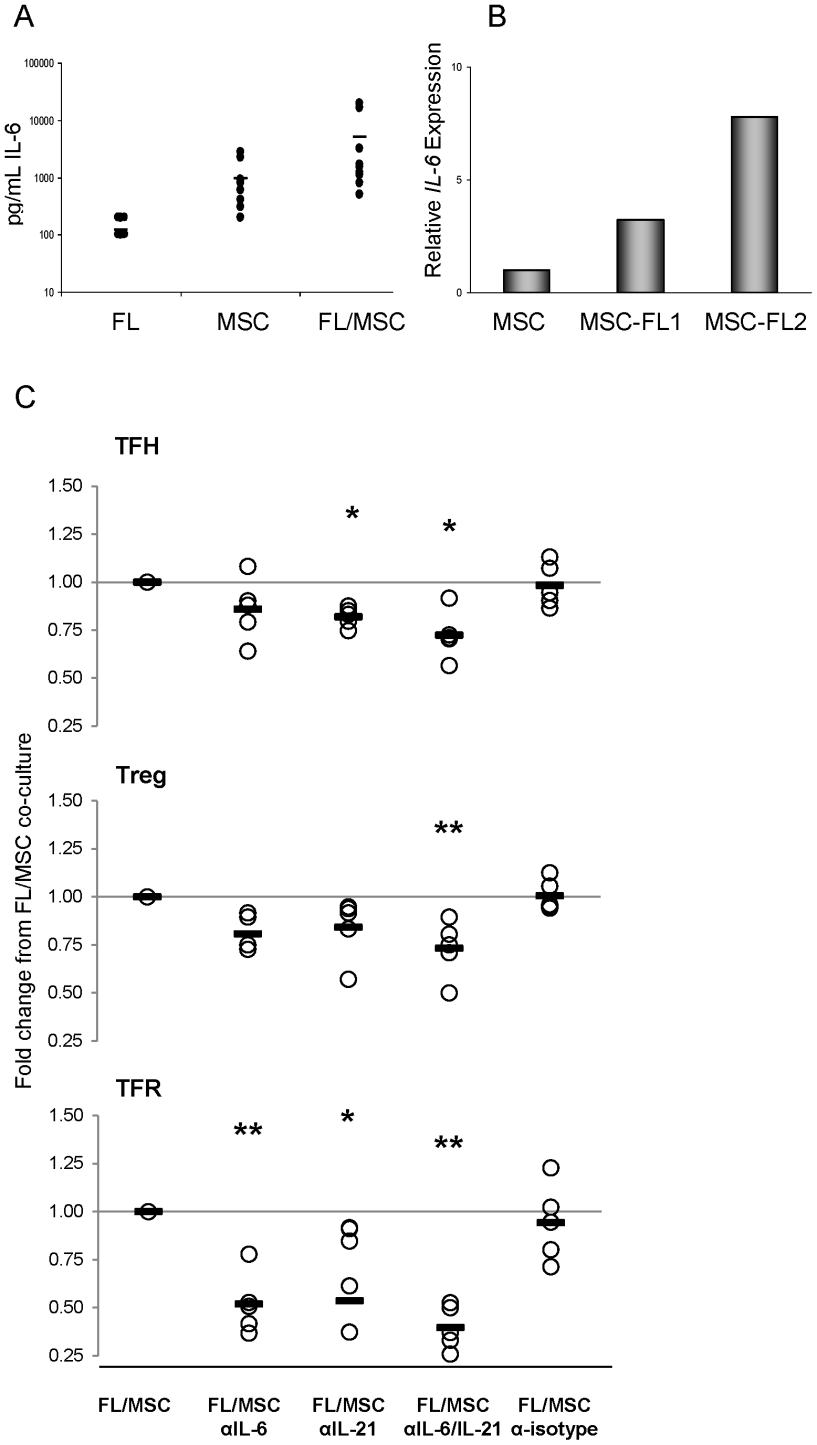
IL-6 and IL-21, in part, support FL-infiltrating T-cell populations. (**A**) Supernatants from 48 hr cultures of FL-SCS, MSCs and FL-SCS/MSCs co-cultures were analyzed for IL-6 by ELISA. The graph is representative of 10 independent cultures from both TN-MSCs and FL-MSCs. (**B**) Quantitative PCR analysis of TN-MSC RNA harvested from isolated TN-MSCs cultured alone or after culture with FL-SCS1 or FL-SCS2. MSC production of IL-6 is enhanced after co-culture with both FL-SCS1 and FL-SCS2. (**C**) Proportions of TFH, Treg and TFR after 48 hr co-cultures with MSCs and neutralizing antibodies specific to IL-6 and/or IL-21 and isotype control, *p<0.05, **p<0.01.

Low levels of IL-6 were detected in the supernatants of FL-SCS (mean 120 pg/mL, n = 8) and TN-MSCs (mean 950 pg/mL, n = 8) when cultured separately for 48 hrs. A greater than additive effect was seen, however, when FL-SCS were co-cultured with TN-MSCs (mean 5100 pg/mL, n = 8) (Fig. 5A). In contrast to that seen with IL-6, IL-21 cytokine levels in the supernatants did not reach the lower limit of detection of our assay. We hypothesized that the increased amount of IL-6 in the FL-SCS and MSC co-cultures was due to cells within the FL-SCS stimulating the MSC to produce IL-6. To address this, TN-MSCs were cultured alone or with FL-SCS. Following 48 hrs of culture the TN-MSCs were isolated and RNA was harvested for quantitative RT-PCR analysis. TN-MSCs cultured alone were compared to TN-MSCs cultured with FL-SCS from subject 1 (FL-SCS1) and TN-MSCs cultured with FL-SCS from subject 2 (FL-SCS2). As shown in Fig. 5B, MSC *IL-6 *mRNA levels were increased, in relation to *GAPDH*, in the MSCs cultured with FL-SCS1 and the MSCs cultured with FL-SCS2 compared to MSCs cultured alone. Specifically, there were 20.79 and 19.75 cycles of *IL-6* in MSCs after co-culture with FL-SCS1 and FL-SCS2, respectively, compared with 22.89 cycles of *IL-6* in the MSCs cultured in media alone. Given that *GAPDH* was consistently 19.8 cycles in all samples, there is approximately a 5.2 and 7.9-fold increase in *IL-6* mRNA in the MSCs cultured with FL-SCS1 and FL-SCS2, respectively, compared to MSCs cultured alone. These data suggest that cells within the FL-SCS induce the expression of *IL-6* mRNA in MSCs.

To determine whether IL-6 played a role in supporting TFH, Treg and TFR cells, we utilized neutralizing antibodies directed against IL-6 in our 48 hr co-culture system. Despite the fact that we did not detect IL-21 in the cultures described above, given the known role that this cytokine plays in TFH development, we also utilized an IL-21 neutralizing antibody. Compared to FL-SCS cultured on MSC the average fold change in TFH after the addition of anti-IL-6, anti-IL-21, the combination of anti-IL-6 and anti-IL-21 or the isotype control antibody to FL-SCS cultured with MSC was 0.86, 0.82, 0.72 and 0.98, respectively (p = 0.489, p = 0.020, p = 0.011, p = 0.463). The addition of anti-IL-6, anti-IL-21, the combination or isotype control resulted in average fold changes to the Treg populations of 0.81, 0.84, 0.73 and 1.01 respectively (p = 0.055, p = 0.106, p = 0.003 and p = 0.557). Finally the addition of antibodies to IL-6, IL-21, the combination of IL-6 and IL-21 or isotype control to FL-SCS cultured with MSC resulted in average fold changes to the TFR populations of 0.52, 0.53, 0.40 and 0.94 respectively (p = 0.001, p = 0.015, p<0.0001, p = 0.457) compared to FL-SCS and MSC cultured without antibodies. A representative example of the flow cytometric plots of these experiments is shown in Figure S2. Taken together, MSC support for TFH, Treg and particularly for TFR appears to be mediated in part by IL-6 and IL-21.

### MSCs Induce Expression of FoxP3 in TFH

**Figure 6:**
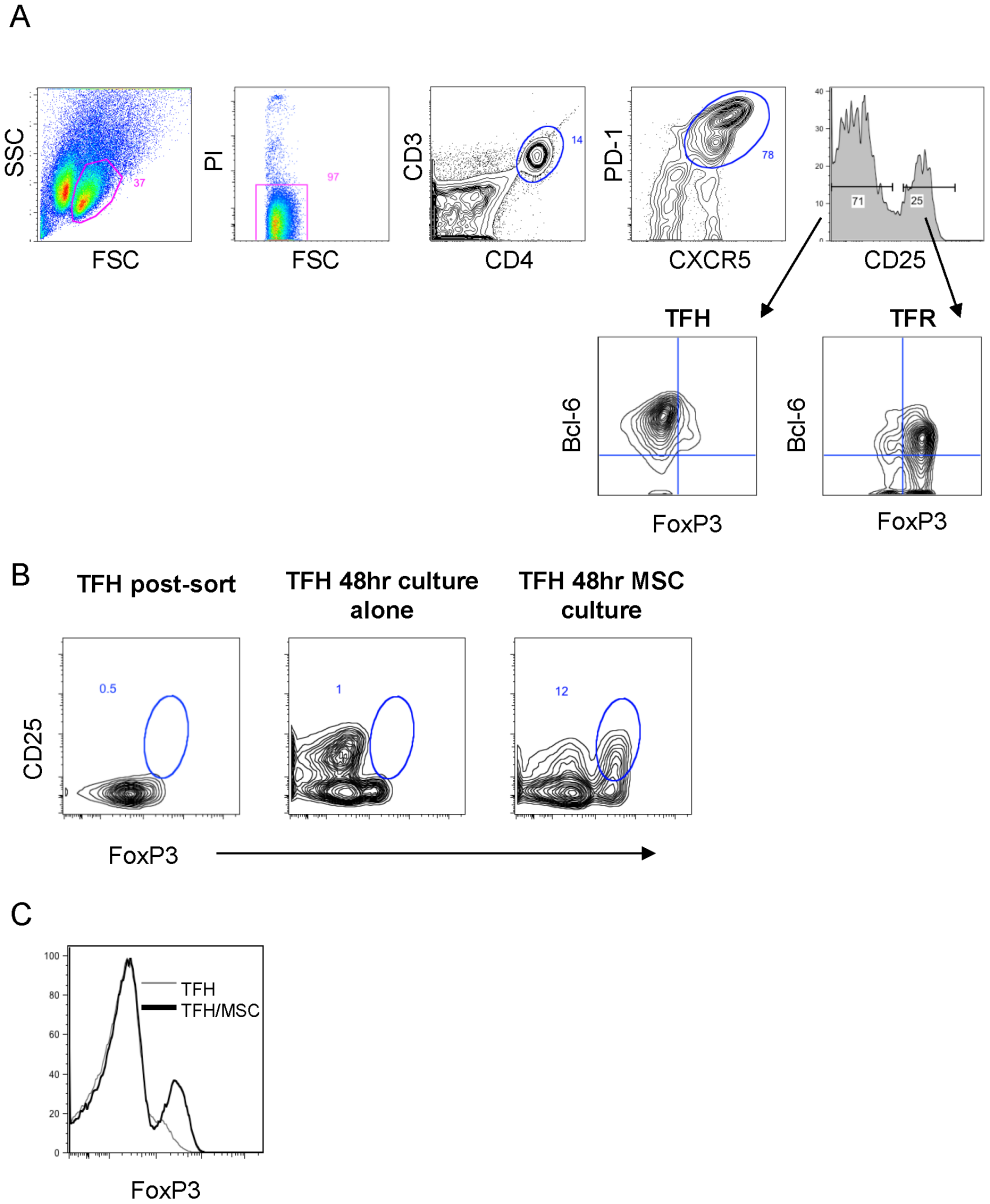
MSCs induce FoxP3 expression in TFH subsets. (**A**) Flow cytometry gating scheme used to isolate TFH cells and post-sort analysis showing Bcl-6 and FoxP3 expression in the isolated cells. (**B**) The percentage of isolated TFH cells expressing CD25 and FoxP3 (TFR) immediately after sorting, at 48 hrs after culture alone and at 48 hrs after co-culture with TN-MSCs. (**C**) Flow cytometry histogram showing FoxP3 expression in TFH after 48 hrs in culture alone and after 48 hrs in culture with TN-MSCs.

MSCs have previously been shown to induce the differentiation of naïve T-cells to Tregs by upregulating the expression of FoxP3 in naïve T-cells. We therefore hypothesized that MSCs would induce the conversion of TFH cells to TFR cells by inducing the expression of FoxP3 in the TFH cells. To test this hypothesis CD3^+^CD4^+^CXCR5^+^PD-1^+^CD25^−^ TFH were isolated from FL-SCS by flow cytometric sorting (Fig. 6A). This phenotype was chosen as CD25 expression discriminates between cells that are phenotypically consistent with TFH and TFR, by virtue of their expression of FoxP3 (CD25^−^Bcl-6^+^FoxP3^−^). A small proportion of the sorted cells were analyzed for intracellular expression of Bcl-6 and FoxP3 while the remaining cells were placed in culture.

The isolated CD3^+^CD4^+^CXCR5^+^PD-1^+^CD25^−^Bcl-6^+^FoxP3^−^ TFH cells were cultured for 48 hr with or without MSCs. The proportion of these cells that expressed CD25 and FoxP3, and had the TFR phenotype, CD3^+^CD4^+^CXCR5^+^PD-1^+^CD25^+^Bcl-6^+^FoxP3^+^, after 48 hrs in culture without MSCs was 1% compared to 12% when the same TFH cells were cultured with MSCs for 48 hrs (Fig. 6B). These results were confirmed in a second independent experiment using a different FL sample. In addition to increasing the proportion of TFH cells that co-expressed CD25 and FoxP3, MSC interactions resulted in increased expression of the FoxP3 protein as evidenced by an increase in the MFI for FoxP3 (Fig. 6C). Taken together, this data supports the hypothesis that a proportion of TFR are derived from TFH and that interactions with MSCs can support this differentiation.

## Discussion

The impact of the tumor stroma on tumor biology is well appreciated [18]–[21]. However, whereas the effects of MSCs on normal and malignant B-cells as well as on Tregs, TH1, TH2 and TH17 cells has been well described, to our knowledge the interplay between human MSCs, TFH and TFR has not been defined. In the experiments described herein, we define TFR as CXCR5^+^PD-1^+^CD25^+^FoxP3^+^Bcl-6^+^ cells, in contrast to the CXCR5^+^PD-1^+^CD25^+^FoxP3^+^ phenotype most often used that does not include Bcl-6. This more stringent characterization was used to separate TFR cells from other regulatory cells that may have gained access to the germinal center but do not necessarily express Bcl-6. In addition, we defined TFH as CXCR5^+^PD-1^+^CD25^−^Bcl-6^+^ cells to exclude the CD25^+^ TFR cells from the TFH population. Finally, we utilized un-manipulated FL-SCS without the addition of exogenous activating factors to more closely recapitulate *in vivo* conditions.

Our data demonstrate that MSCs support the survival of TFH, Treg and TFR by decreasing the proportion of cells that undergo apoptosis. This is indicated by our flow cytometric data which showed there to be a decrease in the percent of active caspase-3 and a greater number of TFH, Treg and TFR viable cells in the MSC co-cultures as compared to that seen when these cells were cultured without MSC. It is important to note that these cells were not proliferating, as no growth factors were added. As such we hypothesize that the MSCs play a greater role in maintaining viability and promoting differentiation rather than in promoting the expansion of these populations. Due to technical challenges associated with generating autologous MSCs from each FL sample studied, we chose to use a consistent MSC population generated from a single tonsillectomy sample. When we compared the findings of MSCs derived from allogeneic TN, allogeneic FL BM (Fig. 2A, 2C) and autologous FL BM or LN, each of the MSC types consistently supported TFH, Treg and TFR viability. We are cognizant of the fact that differences in MSC gene signatures have been demonstrated between MSCs derived from healthy donors and patients with FL [22]. Such differences were attributed, however, to the interactions of the MSCs with the malignant B-cells, suggesting that healthy donor and FL-MSCs are functionally similar and that their biology is ultimately dictated by their interactions with the microenvironmental cells. Taken together, this data supports the use of the allogeneic TN-MSCs for the studies conducted.

MSCs provided T-cell support through soluble factors as evidenced by the increase in the number of TFH, Treg and TFR populations seen when co-cultured in transwell plates with MSC compared to that seen in transwell plate cultures without MSCs. The magnitude of this support was less than that observed in co-cultures providing direct contact, indicating that although soluble factors alone can support these populations, interactions involving both soluble and contact-dependent factors are likely required for optimal support, as has been shown for MSC regulation of other lymphocyte populations [23]–[26].

It is well documented that MSCs can support B-cell viability and that B-cells are capable of providing survival signals to T-cells, including signals that induce the expression of Bcl-6 [27]. In the absence of such B-cell interactions or cytokine support, T-cells will undergo apoptosis *in vitro*. This is particularly true for TFH, which are short-lived and require Bcl-6 activation to maintain CXCR5 expression [28]. While little is known about the signals that are required to promote the longevity and viability of TFH, several factors are thought to be important for their regulation, including TCR ligation, IL-4, IL-21, CD40L, PD-1 ligands and ICOS-ligand [29], [30]. As several of these proteins are expressed by B-cells it is plausible that one mechanism by which MSCs support TFH cells is by supporting B-cells. However, our experiments with CD19^+^ B-cell depleted FL-SCS and flow cytometry sorted CD3^+^CD4^+^ T-cells suggest that B-cells are not required for MSC support of TFH cells, supporting the notion that MSCs can directly support the viability of FL-infiltrating TFH cells. Our data further demonstrates that MSCs can also directly support the viability of FL-infiltrating TFR cells. In contrast, given that there was a significant interaction between MSC and B-cell depletion or (T-cell isolation, Figure 4 A and B, respectively) suggests that the MSC support for Tregs may be influenced by the MSC support of B-cells and/or other cell types, however the small number of samples studied make this only speculative.

Bcl-6 is regulated, in part, by IL-6 a pleiotropic regulatory cytokine produced by several cell types including MSCs. We hypothesized that MSCs support TFH viability through their production of IL-6. The data shown in Fig. 5 support this hypothesis and further demonstrate that MSC-derived IL-6 supports TFR and Treg cell viability. IL-21 similarly regulates Bcl-6, therefore the effect of IL-21 on TFH viability was also determined. Although blocking IL-21 alone did not consistently lead to a significant reduction in TFH, Treg or TFR viability, blocking IL-6 and IL-21 simultaneously reduced TFH, Treg and TFR viability by a statistically significant margin. Although we were unable to detect secreted IL-21 or *IL-21* mRNA in MSCs, it is possible that MSC-derived IL-6 may have stimulated low level production of IL-21 from the T-cell populations and that blocking this in addition to IL-6, resulted in an augmented reduction in the viability of these T-cell subsets compared to blocking IL-6 alone. Interestingly, we found that MSCs co-cultured with FL-SCS had a significant increase in the level of *IL-6* mRNA and secreted proteins compared to that seen with MSCs cultured alone suggesting that FL B-cells and/or another cell population(s) within the microenvironment elicits the generation of IL-6 from MSCs.

Murine TFR populations have been shown to be derived from thymic FoxP3^+^ precursor cells that have co-opted the Bcl-6-driven TFH pathway, however less is known about their origins in human settings [14]–[16]. The ability of MSCs to induce FoxP3 expression in T-cells is well documented. Therefore, we hypothesized and demonstrated that MSCs induce the differentiation of a population of cells that have a TFR phenotype (CD3^+^CD4^+^CXCR5^+^PD-1^+^Bcl-6^+^CD25^+^FoxP3^+^) from purified TFH (CD3^+^CD4^+^CXCR5^+^PD-1^+^Bcl-6^+^CD25^−^FoxP3^−^) cells. Due to the limited size of the FL biopsies obtained and the difficulty of isolating TFR cells after co-culture with MSCs, we were unable to recover a sufficient number of MSC-induced TFR for functional studies. However, the phenotype of these cells is consistent with the current definition of TFR cells and is identical to that of FL-derived TFR that have been shown to functionally inhibit effector T-cells in a previous study [10].

In summary, we demonstrate that MSCs support the viability of TFH and TFR cells. Furthermore, we show that MSCs support the viability of these populations, in part, through the paracrine production of IL-6. Finally, we demonstrate for the first time that TFR can be derived from TFH after co-culture with MSCs. As TFH have been shown to support FL B-cell viability, it would be anticipated that TFR would decrease FL B-cell viability by inhibiting TFH cells. Given our findings that MSCs play a role in TFH and TFR survival and differentiation, we add yet another dimension to the tumor-modulating properties of MSCs in FL, which further suggests its attractiveness as a novel target for therapeutic manipulation.

## Supporting Information

Figure S1 **Representative flow cytometry analysis of TN-derived MSC.** Cells displayed markers characteristic of MSCs (**A**). Oil Red O stain of MSCs cultured in adipocyte differentiation medium and fast red naphthol AS-MX phosphate stain of MSCs cultured in osteoblast differentiation medium showing multipotent differentiation potential of MSCs (**B**).
(TIFF)

Figure S2 **Flow cytometry density plots depicting TFH, Treg and TFR populations within FL-SCS cultured on MSC with the addition of antibodies to IL-6 and/or IL-21 or isotype control antibody.** The percentage of cells within the parent gate is shown, followed by the percentage of the total CD3^+^CD4^+^ T-cells in parentheses.
(TIFF)

Table S1 **Data table displaying the percent of CD3^+^CD4^+^ T-cells characteristic of TFH, Treg and TFR after 48hrs in culture alone or in culture with TN-derived MSC (A) or FL BM-derived MSC for 48 hrs (B).**
(DOCX)
