# Supplementary figures and images for: Mesenchymal Stromal Cells Support the Viability and Differentiation of Follicular Lymphoma-Infiltrating Follicular Helper T-Cells
Source: PLoS One. 2014 May 16;9(5):e97597. doi: 10.1371/journal.pone.0097597 (PMC4023957; doi:10.1371/journal.pone.0097597)

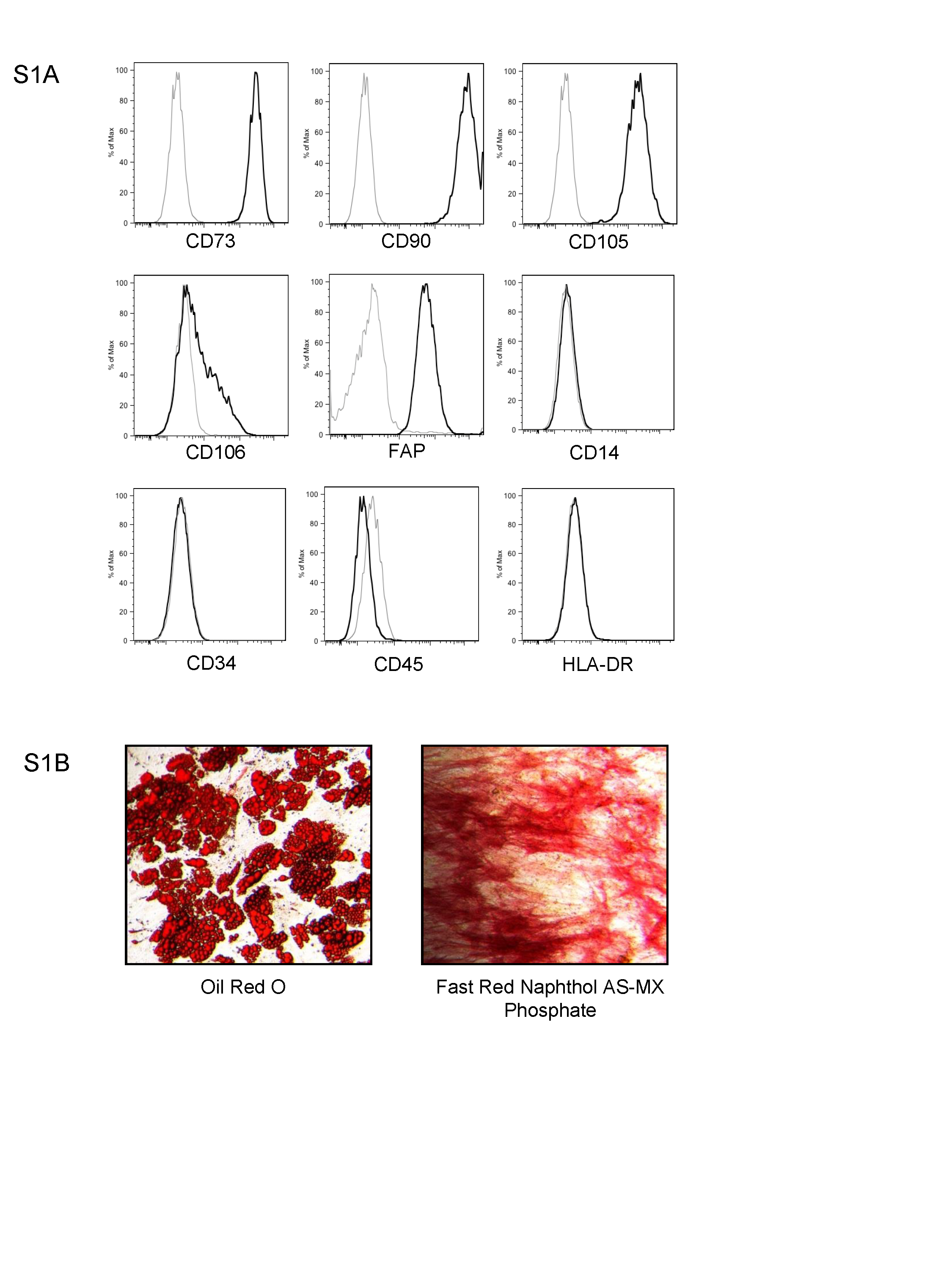

Supplement: Figure S1 — Representative flow cytometry analysis of TN-derived MSC. Cells displayed markers characteristic of MSCs (A). Oil Red O stain of MSCs cultured in adipocyte differentiation medium and fast red naphthol AS-MX phosphate stain of MSCs cultured in osteoblast differentiation medium showing multipotent differentiation potential of MSCs (B). (TIFF) [file pone.0097597.s001.tiff]

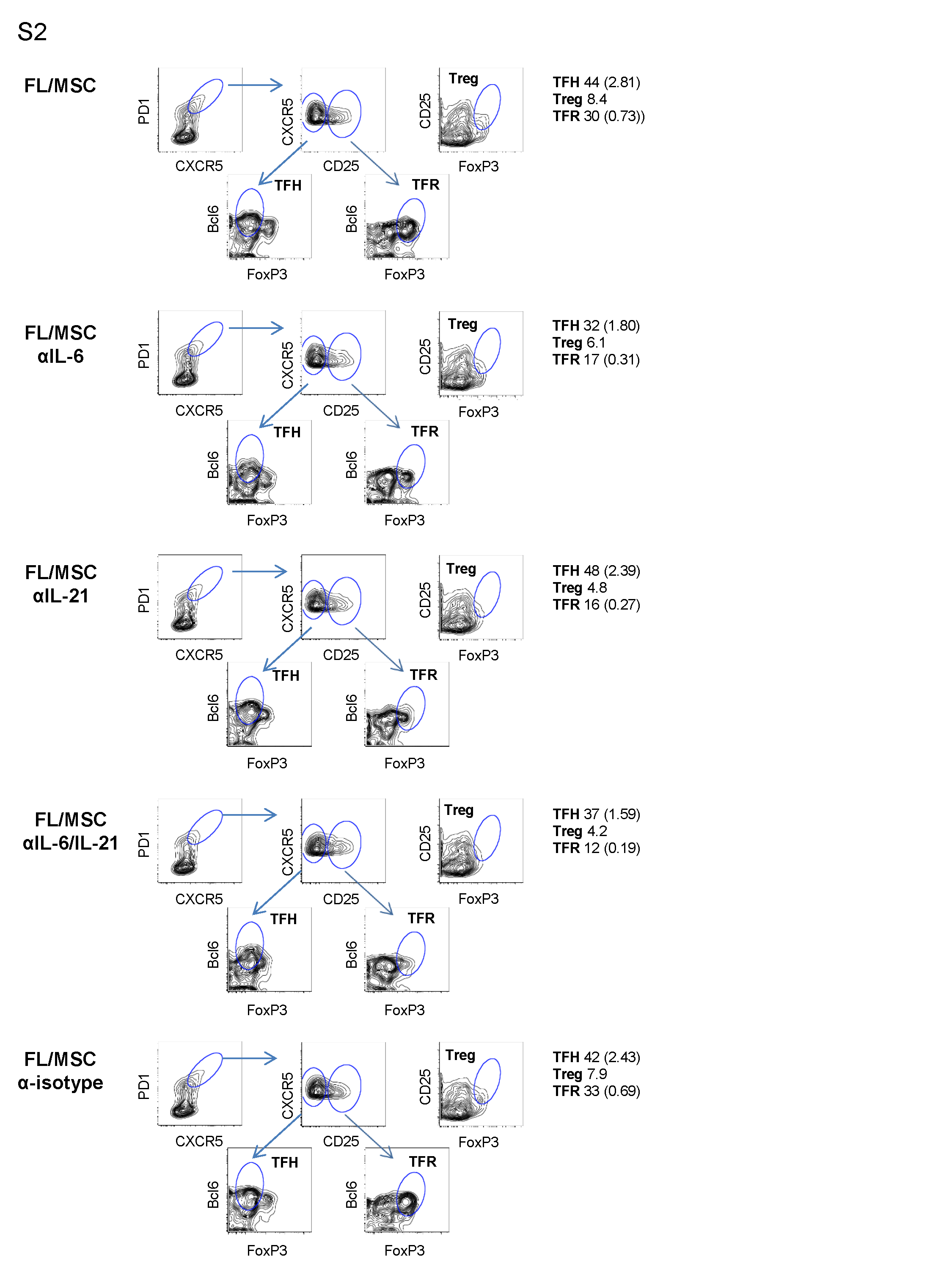

Supplement: Figure S2 — Flow cytometry density plots depicting TFH, Treg and TFR populations within FL-SCS cultured on MSC with the addition of antibodies to IL-6 and/or IL-21 or isotype control antibody. The percentage of cells within the parent gate is shown, followed by the percentage of the total CD3+CD4+ T-cells in parentheses. (TIFF) [file pone.0097597.s002.tiff]
