# Supplementary material for: Mesenchymal Stromal Cells Support the Viability and Differentiation of Follicular Lymphoma-Infiltrating Follicular Helper T-Cells
Source: PLoS One. 2014 May 16;9(5):e97597. doi: 10.1371/journal.pone.0097597 (PMC4023957; doi:10.1371/journal.pone.0097597)
Supplement: Table S1 — Data table displaying the percent of CD3+CD4+ T-cells characteristic of TFH, Treg and TFR after 48hrs in culture alone or in culture with TN-derived MSC (A) or FL BM-derived MSC for 48 hrs (B). (DOCX) [file pone.0097597.s003.docx]

Supplementary Table S1

T-cell subsets cultured with/without MSC (data presented as the % of CD3+CD4+ T-cells)

**1A. TN-derived MSC**

| **Sample ID** | **TFH** | **TFH/MSC** | **Treg** | **Treg/MSC** | **TFR** | **TFR/MSC** |  |  |  |  |  |  |  |  |
| --- | --- | --- | --- | --- | --- | --- | --- | --- | --- | --- | --- | --- | --- | --- |
| 12TB0313 | 8.65 | 13.8 | 8.1 | 13 | .2 | 1.382 |  |  |  |  |  |  |  |  |
| 11TB0596 | 4.24 | 11.4 | 3.1 | 6.5 | 0.017 | 0.322 |  |  |  |  |  |  |  |  |
| 11TB0596 | 28.81 | 34.84 | 17 | 28 | 1.39 | 2.428 |  |  |  |  |  |  |  |  |
| 11TB0596 | 3.33 | 6.13 | 1.6 | 4.8 | .074 | .594 |  |  |  |  |  |  |  |  |
| 11TB0596 | 1.24 | 2.79 | 1.3 | 3 | .031 | .419 |  |  |  |  |  |  |  |  |
| 11TB0596 | 5.27 | 12.32 | 4.8 | 8.2 | .216 | 1.882 |  |  |  |  |  |  |  |  |
| 11TB0500 | 17.63 | 19.41 | 15 | 21 | .297 | .658 |  |  |  |  |  |  |  |  |
| 11TB0112 | 9.11 | 16.18 | 2 | 3.5 | .016 | .108 |  |  |  |  |  |  |  |  |
| 11TB0112 | 19.51 | 24.4 | 2.6 | 4.7 | .013 | .109 |  |  |  |  |  |  |  |  |
| 11TB0112 | 12.49 | 16.9 | 4.2 | 6.9 | .034 | .122 |  |  |  |  |  |  |  |  |
| 11TB0112 | 7.83 | 14.29 | 1.7 | 3.5 | 0.02 | .151 |  |  |  |  |  |  |  |  |
| 11TB0084 | 2.03 | 4.26 | 5.7 | 13 | .084 | .817 |  |  |  |  |  |  |  |  |
| 11TB0080 | 20.4 | 28.83 | 5.2 | 15 | .269 | 3.724 |  |  |  |  |  |  |  |  |
| 11TB0134 | 8.54 | 12.77 | 4.7 | 12 | .347 | 1.109 |  |  |  |  |  |  |  |  |
| 11TB0134 | 5.38 | 11.14 | 2.2 | 11 | 0.591 | 5.481 |  |  |  |  |  |  |  |  |
| 11TB0134 | 7.39 | 12.77 | 4.8 | 10 | 0.319 | 1.003 |  |  |  |  |  |  |  |  |
| 09TB0063 | 8.15 | 16.82 | 11 | 20 | .109 | .678 |  |  |  |  |  |  |  |  |
| 12TB0141 | 3.86 | 6.34 | 15 | 26 | 1.410 | 3.713 |  |  |  |  |  |  |  |  |
| 11TB0217 |  |  | 14 | 17 |  |  |  |  |  |  |  |  |  |  |
| 11TB0217 |  |  | 9.8 | 15 |  |  |  |  |  |  |  |  |  |  |
| 11TB0084 |  |  | 1.6 | 7 |  |  |  |  |  |  |  |  |  |  |
| 11TB0500 |  |  | 10 | 17 |  |  |  |  |  |  |  |  |  |  |

**Table 1B. FL BM-derived MSC**

| **Sample ID** | **TFH** | **TFH/FL-MSC** | **Treg** | **Treg/FL-MSC** | **TFR** | **TFR/FL-MSC** |
| --- | --- | --- | --- | --- | --- | --- |
| 11TB0112 | 14.17 | 18.46 | 1.7 | 3.4 | 0.072 | 0.107 |
| 10TB0006 | 4.57 | 7.71 | 8.4 | 11 | 0.026 | 0.119 |
| 11TB0072 | 5.16 | 5.85 | 18 | 23 | 0.349 | 0.92 |
| 11TB0084 | 1.58 | 2.46 | 2 | 2.3 | 0.059 | 0.318 |
| 11TB0112 | 8.88 | 13.39 | 0.7 | 1.7 | 0.031 | 0.039 |
| 11TB01134 | 5.27 | 9.34 | 2.2 | 4.4 | 0.175 | 0.504 |
| 11TB0596 | 3.26 | 6.94 | 0.8 | 1.7 | 0.124 | 0.23 |
